# Cephalometric measures correlate with polysomnography parameters in individuals with midface deficiency

**DOI:** 10.1038/s41598-021-85935-7

**Published:** 2021-04-12

**Authors:** Renato Fortes Bittar, Sílvio Eduardo Duailibi, Gabriela Pereira Ribeiro Prado, Lydia Masako Ferreira, Max Domingues Pereira

**Affiliations:** 1 Postgraduate Program in Translational Surgery, Federal University of Sao Paulo, Rua Botucatu 740, 2nd floor, Vila Clementino, São Paulo, SP CEP 04023-900 Brazil; 2 Division of Plastic Surgery, Department of Surgery, Federal University of Sao Paulo, São Paulo, SP Brazil

**Keywords:** Sleep disorders, Risk factors

## Abstract

To determine the association between cephalometric measurements and polysomnographic parameters in Brazilian patients with midface deficiency. This was a primary, clinical, observational, longitudinal, retrospective, analytical, and single-center study. Forty-eight patients with midface deficiency were divided into two groups as follows: those who underwent surgically assisted rapid palatal expansion (SARME) and those who received maxillary advancement (MA). Pre- and post-operative cephalometric and polysomnography measurements were obtained. Pearson's correlation was used to verify the presence of any significant associations between PSG scores and cephalometric measurements. Associations between BMI (Body Mass Index) and AHI (Apnea Hypopnea Index) as well as arousals were observed. In the SARME group, associations between AHI and SNA, UAS and MP-H, arousals and SNA, and Co-A and MP-H were noted. Associations between AHI and Co-A, PoOr-A and MP-H, arousals and UAS, and between minimum saturation of O2 and SNA, SNB, and Co-A were observed in the MA group. This study demonstrates the alterations in the middle third of the face that were related to sleep disturbance. In addition, it shows the associations between the polysomnographic parameters and the cephalometric representations corresponding to the analyzed deformities and transverse or anteroposterior maxillary deficiencies.

## Introduction

Deformities of the craniofacial skeleton may be congenital or acquired in nature. Those that affect the mandible, maxilla or dentition are called dentofacial deformities (DFD)^1^.

The degree of the DFD may vary, ranging from slight asymmetries without functional and/or aesthetic repercussions to significant changes that affect the entire functioning of the stomatognathic system, and sometimes, the respiratory system^2^. There is a close relationship between DFD and sleep-disordered breathing, and it is estimated that several individuals with DFD who are candidates for orthognathic surgery experience obstructive sleep apnea syndrome (OSAS)^3^.

Deformities of the lower third of the face are more-commonly associated with OSA. However, the association of midface facial DFD with OSA was initially proposed in 1984; this prompted an interest in studying the relationship between maxillary deformities and OSAS^4–7^. The deficiency of the middle third of the face comprises three-dimensional changes that generate changes in the height, width, and anteroposterior relationship of the maxilla. These changes occur in both syndromic and non-syndromic individuals with repercussions on dental occlusion, phonation, and nasal breathing^8,9^. Changes in the anteroposterior and transverse dimensions of the maxilla cause narrowing of the nasal cavities and alterations in the base of the skull, which lead to a reduction in the nasopharynx airspace^10^. Retro positioned maxilla can encroach the pharyngeal airway and the narrow transverse dimension increases the airflow resistance^11^.

Initially used as an exam for the diagnosis of craniofacial deformities, cephalometry is currently used in sleep medicine to correlate craniofacial changes with the presence of OSAS^3^. Following the association between craniofacial changes and the etiology of upper airway obstruction, several studies attempted to determine the anatomical abnormalities that might be most closely correlated; mandibular deficiency, cranial base hyperflexion, and transverse and sagittal deficiencies of the maxilla and mandible were found to be the most important^7,12,13^. The hyoid bone, which provides attachment to the muscles of the floor of the mouth, is found at a lower position in patients with OSAS which leads to an increment in the MP-H (Mandibular plane to Hyoid) distance^14^.

Thus, cephalometric measurements have been studied to demonstrate a correlation with the diagnosis and severity of OSAS, such as the presence of a value above the normal distance between the posterior nasal spine and the distal end of the uvula (PNS-P) and the distance between the mandibular plane and the hyoid bone (MP-H), both correlating with altered apnea and the hypopnea index (AHI)^15–18^. About 86% of OSAS patients are estimated to have reduced retrolingual airspace^19^.

Cephalometry has been used to evaluate the success of the OSAS treatment, and some studies have suggested that SNA (angle formed between the point A, N and S), lower facial height, and palate thickness may act as variables^20–22^. Therefore, considering the high prevalence of individuals with DFDs, particularly, sagittal and transverse maxillary deficiencies, in the population, and the fact that this condition is often associated with the presence of OSAS^8,9^, this study aimed to determine the association between cephalometric measurements and the polysomnographic parameters in Brazalian patients with midface deficiency.

## Materials and methods

### Study design

This is a primary, clinical, observational, longitudinal, retrospective, analytical, and single-center study.

### Ethical considerations

The study was submitted and approved by the Research Ethics Committee of the Federal University of São Paulo (UNIFESP; approval number, 0178/2017).

#### Declarations

All the phases of the study strictly followed the Declarations of Helsinki for experiments involving humans. Informed consent was obtained from all the participants in this study and also obtained from a parent and/or legal guardian of under 18 years patient.

### Patient selection

Medical records and exams of patients operated in the cranio-maxillofacial surgery department of the Plastic Surgery Discipline of the Federal University of São Paulo (UNIFESP), from 2002 to 2018.

#### Inclusion criteria

Patients with transverse or sagittal maxillary deficiency, with or without OSAS, who underwent surgically assisted rapid palatal expansion (SARME) or maxillary advancement (MA) were included in this study.

#### Criteria for non-inclusion

The following patients were excluded from the study: those with any syndromes affecting craniomaxillofacial development; those with incomplete cephalometric, polysomnographic and operative documentation; and those undergoing any other surgical modality concomitantly.

#### Sample

After selecting the medical records, 48 patients were divided into two groups according to the type of surgery performed: Group 1, patients who underwent SARME (n = 25; Table 1) and Group 2, those who underwent MA (n = 23; Table 2). SARME was indicated when the transverse maxillary discrepancy was severe for correction with orthodontic approach only. Similarly, maxillary advancement was done in patients where the discrepancy was severe in sagittal dimension and beyond the scope of orthodontic camouflage. The severity of the discrepancy was assessed clinically and radiographically (cephalometry).

**Table 1:** Characteristics of patients selected from the SARME group.

Patient	Age	BMI	Mov	AHI	Arousals	MinSatO2	TST sat < 90%
1	31	24.51	8.7	3	0	89	3.2
2	26	27.1	8	0.9	0	94	0
3	24	28.52	9.2	0.2	12	69	0.3
4	24	17.19	8.7	1.3	0	89	0.1
5	46	34.15	8.5	13.4	0	85	0.4
6	33	22.59	7.8	0.4	14	95	0
7	37	25.91	7	18.3	6.9	61	6.1
8	40	29	9	8.1	25	91	0
9	30	27.94	8.3	3	0	86	3.2
10	47	36.73	8	13.2	15	71	3.1
11	37	21.3	8.2	0	0	96	0
12	26	29	7	0.1	8	95	0
13	16	28.9	7.5	0.3	12	92	0
14	26	22.84	11.3	1.2	0	92	0
15	29	18.38	8.4	0.4	0	93	0
16	32	25.2	9	2	9	92	0
17	34	22	9	1	9.9	97	0
18	19	27.6	7.7	2.6	7	95	0
19	30	22.72	10.2	0.2	0	93	0
20	29	38.06	11.2	44.8	42.7	85	4.3
21	34	24.38	8.8	6.5	0	67	3.8
22	32	29.06	7.4	0	0	91	0
23	26	22.1	7.2	0	4.4	93	0
24	25	30.1	10.1	20.7	6	76	1.3
25	25	25.97	7.4	0.3	2.7	93	0
Average	30.32	26.45	8.54	5.68	6.98	87.2	1.03
SD	7.35	5.06	1.18	10.13	9.87	10.14	1.77

Age: years; BMI: kg/m^2^; Mov. Millimeters; AHI: number per hour; Arousal: number per hour; MinSat^O2^: percentage; TST sat < 90%: percentage of the total time of sleep; SD: standard deviation.

**Table 2:** Characteristics of the patients in the MA group.

Patient	Age	BMI	Mov	AHI	Arousals	minSatO2	TST sat < 90%
1	45	28	5	10.7	17	90	0
2	19	24.22	7	0.2	25	97	0
3	30	29.05	6	2.4	2.4	95	0
4	18	22.4	5	0.6	18	94	0
5	39	26.08	6.5	2.1	13	86	3.2
6	20	26.22	10	0.2	0.4	95	0
7	34	25.01	4	0.5	4.7	93	0
8	24	27.2	9	0	7.5	94	0
9	37	27.82	8.5	2.2	7.8	85	1.2
10	47	26.4	6	0	5.7	92	0
11	34	25.4	10	18	45	91	0
12	23	19.8	9	0	15	98	0
13	31	29.06	7	11.2	22.2	84	0.5
14	44	27.1	7.5	4	21.2	95	0
15	37	25.07	5	0	2.5	97	0
16	31	28.04	6	11.4	13.1	88	2.5
17	31	27.4	8	0.5	0	91	0
18	25	28.01	8	0	6.9	92	0
19	40	31.22	5	0	7.9	94	0
20	24	22.49	9	1.7	8.2	93	0
21	17	25.4	8	0	4.8	94	0
22	21	23.85	5	0	0	97	0
23	49	24.02	7	0	11.2	97	0
Average	31.3	26.05	7.02	2.86	11.28	92.7	0.32
SD	9.79	2.56	1.75	4.96	10.34	3.94	0.85

Age: years; BMI: kg/m^2^; Mov. Millimeters; AHI: number per hour; Arousal: number per hour; MinSatO2: percentage; TST sat < 90%: percentage of the total time of sleep; SD: standard deviation.

Lateral cephalograms were performed in all the patients at two different periods: T1 (preoperative) and T2 (6 months after surgery). Teleradiographs were scanned at a resolution of 400 dpi (dots per inch), filed in the JPEG (Joint Photographic Experts Group) format, and edited using the public domain ImageJ software (Wayne Rasband's ImageJ) developed by the National Institute of Health.

The following cephalometric landmarks were defined: Sella (S), point located at the geometric center of the Sella turcica image; Nasion (N), point located in the most anterior region of the frontal-nasal suture, meeting the profile lines of the glabella and nasal bones; Orbital (Or), the lowest point of orbit contour; Porion (Po), point located on the upper and outer margin of the external ear canal; Subspinatus or point A (A), point located at the deepest curve of the alveolar profile of the maxilla; Supramentonian or point B (B), deepest point of anterior mandibular concavity; Mentus (Me), most anterior and inferior point of the bony chin; Gonius (Go), the lowest and posterior point in the gonial region at the mandibular angle; Hyoid (H), the most anterosuperior point of the body of the hyoid bone; Condylum (Co), upper and posterior point of the mandibular condyle; and Tongue base (T1), most posterior point of the tongue base.

**Figure 1:**
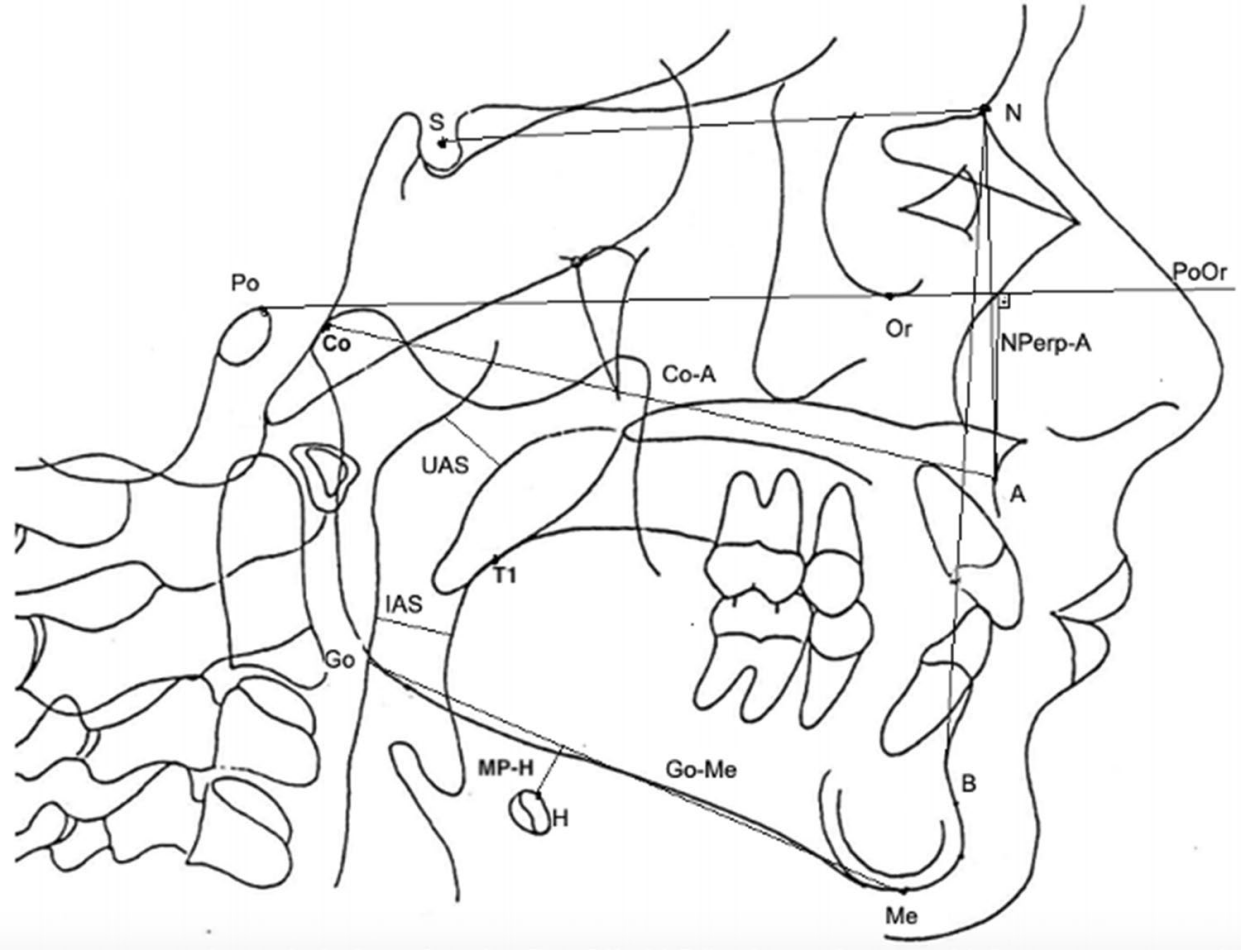
S (Sella); N (Nasion); Or (Orbital); Po (Porion); A (Point A); B (Point B); Me (Mentus); Go (Gonius); H (Hyoid); Co (Condylum); T1 (Tongue base).

Subsequently, the following angular and linear measurements were taken: SNA, the angle formed between points S-N-A; SNB, the angle formed between points S-N-B; effective jaw length, measured between Co-A points; PoOr-A, linear measurement between the line joining Po-Or points and its orthogonal plotted to point A; NPerp-A, distance from point A to a line that passes at point N and perpendicular to the PoOr plane; Upper airspace (UAS), measurement drawn from the posterior wall of the soft palate to the region of the smallest cross-section of the posterior pharyngeal wall; Lower airspace (IAS), measured from the base of the tongue (T1) and the region of the greatest narrowing with the posterior pharyngeal wall; and Hyoid bone height (MP-H), orthogonal distance from the mandibular plane (Go-Me) and the H point (Fig. 1).

Overnight type 1 polysomnography was performed in all patients before the surgery (T1) and postoperatively (T2) at Sleep Institute Clinic, Sao Paulo – Brazil. The patients were accommodated in a dark and quiet room with a comfortable bed. Electroencephalogram (EEG), submental and tibial electromyography, right and left electro-oculogram, electrocardiography, airflow (oral and nasal), respiratory effort (chest and abdomen movements), and oxyhemoglobin saturation (SatO2%) were recorded with finger pulse oximetry. A microphone was used to detect snoring and lying positions. Apnea is considered as complete cessation of breathing for ≥ 10 secs and hypopnea is considered as an abnormal respiratory event for ≥ 10 s with ≥ 30% reduction in airflow with a fall of ≥ 4% of SPO2. AHI consisted of the total number of apnea or hypopneas per hour of sleep.

#### Statistical analysis

All reference points were plotted at three different times with 15 days difference by the same examiner to evaluate intraobserver agreement through the intraclass correlation coefficient (ICC). The average of the values of three measurements was used for each measure. The null hypothesis was that the measurements were not concordant. Pearson’s correlation test was performed to assess the relationship between the cephalometric and polysomnographic measurements for each type of surgery. Scatter plots were used for visual analysis in the case of moderate, strong, and very strong correlations. The classification proposed by DEVORE^23^ was used to estimate the power of the correlation as follows: 0–0.19, very poor correlation; 0.20–0.39, weak correlation; 0.40–0.69, moderate correlation; 0.70–0.89, strong correlation; and 0.90–1.00, very strong correlation.

Initially, the association between the BMI and age of the entire sample (n = 48) with polysomnographic parameters was analyzed. Then, the analysis of the associations in each group was performed.

For significant correlations, the mediation effect in multiple linear regression was tested, analyzing the interaction of BMI and age in the association.

### Research involving human participants and/or animals

The study was submitted and approved by the Research Ethics Committee of the Federal University of São Paulo (UNIFESP; approval number, 0178/2017). All the phases of the study strictly followed the Declarations of Helsinki for experiments involving humans.

### Informed consent

Informed consent was obtained from all the participants in this study. The informed consent was previously analyzed and approved by the Research Ethics Committee of the Federal University of São Paulo (UNIFESP).

## Results

The values of the cephalometric and polysomnographic measurements at T1 and T2 in patients who underwent SARME and MA are shown in supplementary online tables.

### Intraclass correlation coefficient (ICC)

The ICC values were highly significant (p < 0.0001) for all the measurements, thus rejecting the null hypothesis (Table 3).

**Table 3:** Intraclass correlation coefficient (ICC).

Parameter	SNA	SNB	Co-A	PoOr-A	NPerp-A	UAS	IAS	MP-H
**SARME**								
T1	0.999	0.969	0.999	1.000	1.000	0.999	0.999	0.999
T2	0.986	0.99	0.993	0.999	1.000	0.998	0.999	0.998
**MA**								
T1	0.999	0.999	1.000	1.000	1.000	0.996	0.997	0.998
T2	0.992	0.992	0.997	0.998	0.999	0.995	0.996	0.997

T1: preoperative; T2: postoperative.

### Pearson’s correlation

Both age and BMI have a positive correlation with AHI, Arousals and TST SatO2 < 90% whereas they are negatively correlated with Min SatO2 (Table 4). Similar correlations were obtained when these parameters were assessed separately for SARME and MA groups (Tables 5 and 6).

**Table 4:** Pearson’s correlation (entire sample) between BMI and AGE with AHI, arousals, minimum O2 saturation and total sleep time with < 90% O2 saturation.

Entire sample	BMI (p-value)	AGE (p-value)
AHI	0.54 (p = 0.999)	0.19 (p = 0.174)
Arousals	0.37 (p = 0.995)	0.1 (p = 0.478)
Min SatO2	− 0.34 (p = 0.008)	− 0.20 (p = 0.08)
TST SatO2 < 90%	0.35 (p = 0.992)	0.2 (p = 0.74)

N = 48; AHI: Apnea Hypopnea Index; Min SatO2: minimum saturation of O2); TST SatO2 < 90%: total sleep time with saturation of O2 below 90%.

**Table 5:** Pearson’s correlation (SARME) between BMI and AGE with AHI, arousals, minimum O2 saturation and total sleep time < 90% O2 saturation.

SARME	BMI (p-value)	AGE (p-value)
AHI	0.62 (p = 0.0008)	0.24 (p = 0.235)
Arousals	0.66 (p = 0.0003)	0.08 (p = 0.69)
Min SatO2	− 0.34 (p = 0.04)	− 0.29 (p = 0.07)
TST SatO2 < 90%	0.4 (p = 0.04)	0.31 (p = 0.235)

N = 25; AHI: Apnea Hypopnea Index; Min SatO2: minimum saturation of O2); TST SatO2 < 90%: total sleep time with saturation of O2 below 90%.

**Table 6:** Pearson’s correlation (MA) between BMI and AGE with AHI, arousals, minimum O2 saturation and total sleep time with < 90% O2 saturation.

MA	BMI (p-value)	AGE (p-value)
AHI	0.25 (p = 0.233)	0.22 (p = 0.301)
Arousals	− 0.10 (p = 0.310)	0.1 (p = 0.632)
MinSatO2	− 0.49 (p = 0.008)	− 0.25 (p = 0.121)
TSTsatO2 < 90%	0.18 (p = 0.04)	0.16 (p = 0.301)

N = 23; AHI: Apnea Hypopnea Index; Min SatO2: minimum saturation of O2); TST SatO2 < 90%: total sleep time with saturation of O2 below 90%.

For the SARME group, significant associations between AHI, arousals, MinSatO2, < 90% TSTSatO2, and cephalometric measures were observed preoperatively (Table 7). Two polysomnographic measurements (AHI and Arousals) were correlated with some of the cephalometric measurements. Preoperative AHI was significantly correlated with preoperative SNA (r = 0.499/p = 0.011), UAS (r = − 0.488/p = 0.013), and MPH (r = 0.573/p = 0.03) and preoperative arousals were significantly correlated with preoperative SNA (r = 0.410/p = 0.042), Co-A (r = 0.491/p = 0.013), and MPH (r = 0.507/p = 0.010). (Online Supplementary Figures 2–7).

**Table 7:** Pearson’s correlations between AHI, Arousals, MinSatO2, TSTSatO2 < 90% and T1 and T2 cephalometric measures. SARME.

	SNA	SNB	Co-A	PoOr-A	NPerp-A	UAS	IAS	MP-H
**T1**								
AHI	0.499^a^ p = 0.011	0.221 P = 0.289	0.290 p = 0.160	− 0.148 p = 0.481	− 0.365 p = 0.073	− 0.488^b^ p = 0.013	0.092 p = 0.662	0.573^c^ p = 0.03
Arousals	0.410^d^ p = 0.042	0.228 p = 0.274	0.491^e^ p = 0.013	0.039 p = 0.855	− 0.176 p = 0.399	− 0.180 p = 0.389	0.069 p = 0.742	0.507^f^ p = 0.010
MinSatO2	− 0.175 p = 0403	0.209 p = 0.316	− 0.122 p = 0.560	0.093 p = 0.657	0.299 p = 0.146	0.177 p = 0.398	− 0.069 p = 0.744	− 0.320 p = 0.118
TSTSatO2 < 90%	0.284 p = 0.169	0.045 p = 0.830	0.208 p = 0.319	0.043 p = 0.840	− 0.266 p = 0.198	− 0.349 p = 0.087	0.114 p = 0.587	0.356 p = 0.081
**T2**								
AHI	0.378 p = 0.062	0.313 p = 0.127	0.199 p = 0.340	− 0.162 p = 0.438	− 0.204 p = 0.329	− 0.366 p = 0.072	− 0.242 p = 0.244	0.111 p = 0.596
Arousals	0.060 p = 0.777	0.016 p = 0.940	0.246 p = 0.236	0.262 p = 0.206	− 0.196 p = 0.347	− 0.252 p = 0.225	− 0.147 p = 0.482	0.234 p = 0.260
MinSatO2	− 0.283 p = 0.170	− 0.173 p = 0.408	− 0.135 p = 0.521	0.283 p = 0.171	0.248 p = 0.233	0.284 p = 0.169	0.350 p = 0.086	− 0.049 p = 0.816
TSTSatO2 < 90%	0.258 p = 0.214	0.315 p = 0.125	0.105 p = 0.616	− 0.247 p = 0.235	− 0.144 p = 0.492	− 0.323 p = 0.115	− 0.272 p = 0.189	0.033 p = 0.877

Online Supplementary Figures: a-Figure 2; b-Figure 3; c-Figure 4; d-Figure 5; e-Figure 6; f-Figure 7.

**S**ignificant correlations were observed preoperatively in patients who underwent the MA surgical procedure (Table 8). The significant correlations were as follows: preoperative AHI was significantly correlated with preoperative CoA (r = 0.424/p = 0.044), PoOr-A (r = 0.551/p = 0.006), and MPH (r = 0.479/p = 0.021); preoperative arousals were significantly correlated with preoperative UAS (r = − 0.440/p = 0.035); and minimum preoperative O2 saturation was significantly correlated with preoperative SNA (r = − 0.415/p = 0.049), SNB (r = − 0.468/p = 0.024), and CoA (r = − 0.414/p = 0.049). (Online Supplementary Figures 8–14).

**Table 8:** Pearson’s correlations between AHI, Arousals, MinSatO2, TSTSatO2 < 90% and T1 and T2 cephalometric measures. MA.

	SNA	SNB	Co-A	PoOr-A	NPerp-A	UAS	IAS	MPH
**T1**								
AHI	− 0.035 p = 0.876	0.022 p = 0.920	0.424^a^ p = 0.044	0.551^b^ p = 0.006	− 0.315 p = 0.143	− 0.120 p = 0.586	0.206 p = 0.345	0.479^c^ p = 0.021
Arousals	− 0.131 p = 0.553	− 0.118 p = 0.590	− 0.006 p = 0.977	0.159 p = 0.469	0.170 p = 0.439	− 0.440^d^ p = 0.035	− 0.058 p = 0.794	0.066 p = 0.765
MinSatO2	− 0.415^e^ p = 0.049	− 0.468^f^ p = 0.024	− 0.414^g^ p = 0.049	− 0.247 p = 0.255	− 0.118 p = 0.591	− 0.130 p = 0.556	− 0.214 p = 0.326	− 0.188 p = 0.390
TSTSatO2 < 90%	0.159 p = 0.470	0.093 p = 0.672	0.014 p = 0.950	0.038 p = 0.863	0.190 p = 0.386	− 0.003 p = 0.989	0.010 p = 0.964	0.222 p = 0.308
**T2**								
AHI	− 0.042 p = 0.848	0.115 p = 0.600	0.224 p = 0.304	0.405 p = 0.055	0.191 p = 0.382	0.178 p = 0.417	− 0.012 p = 0.957	− 0.042 p = 0.850
Arousals	0.022 p = 0.922	0.111 p = 0.614	0.115 p = 0.600	0.215 p = 0.324	0.005 p = 0.983	− 0.218 p = 0.317	− 0.337 p = 0.116	0.155 p = 0.481
MinSatO2	− 0.058 p = 0.793	− 0.223 p = 0.307	− 0.258 p = 0.235	− 0.469 p = 0.024	− 0.131 p = 0.552	− 0.288 p = 0.183	− 0.142 p = 0.516	0.113 p = 0.607
TSTSatO2 < 90%	0.022 p = 0.922	0.111 p = 0.614	0.115 p = 0.600	0.215 p = 0.324	0.005 p = 0.983	− 0.218 p = 0.317	− 0.337 p = 0.116	0.155 p = 0.481

Online Supplementary Figures: a-Figure 8; b-Figure 9; c-Figure 10; d-Figure 11; e-Figure 12; f-Figure 13; g-Figure 14.

The mediation regression was shown to be significant with p values < 0.05 between the association SNB/arousals and MP-H/AHI in the SARME group when moderated by BMI, with p-values equal to 0.026 and 0.025, respectively.

For the MA group, the mediation regression showed an interaction of the BMI with the SNB/MinSatO2 association, with a p-value = 0.030 (Table 9).

**Table 9:** Mediation effect in multiple linear regressions of the significant associations.

	BMI					
SARME	*SNB/AROUSALS*	SQ	DF	MQ	Z	p-value
	Regression	8.423	3	2.808	3.785	0.026
	Residuals	15.577	21	0.742		
	Total	24	24			
SARME	*MPH/AHI*					
	Regression	8.444	3	2.815	3.8	0.025
	Residuals	15.556	21	0.741		
	Total	24	24			
MA	*SNB/MinSatO2*					
	Regression	8.101	3	2.7	3.692	0.03
	Residuals	13.899	19	0.732		
	Total	22	22			

SQ: sums of the squares; DF: degrees of freedom; MQ: means of squares; Z: Z-score.

There was no interaction between the AGE variable and the significant associations.

## Discussion

This study was conducted to explore the association between the cephalometric measures and polysomnographic parameters in individuals with midface deficiency. Two polysomnographic parameters- pre-operative AHI and arousals were found to be associated with different cephalometric measures. Preoperative AHI was significantly correlated with preoperative SNA (r = 0.499/p = 0.011), UAS (r = − 0.488/p = 0.013), and MPH (r = 0.573/p = 0.03) in the SARME group and CoA (r = 0.424/p = 0.044), PoOr-A (r = 0.551/p = 0.006), and MPH (r = 0.479/p = 0.021) in the MA group. Similarly, pre-operative arousals were significantly associated with SNA (r = 0.410/p = 0.042), Co-A (r = 0.491/p = 0.013), and MPH (r = 0507/p = 0.010) in the SARME group and SNA (r = − 0.415/p = 0.049), SNB (r = − 0.468/p = 0.024), and CoA (r = − 0.414/p = 0.049) in the MA group.

Cephalometry does not have the technical resources to provide the data necessary to reach a diagnosis of OSAS because it involves the identification and quantification of both apnea and hypopnea. However, the identification of craniofacial changes detectable by cephalometry may help in the propaedeutic arsenal, providing additional information about individuals more prone to OSAS, thus helping to rationalize the use of complementary exams.

Polysomnography is considered as the gold standard for the diagnosis of OSAS, however, this is a complex, uncomfortable and expensive procedure and hence not routinely performed before orthognathic surgery for the correction of dentofacial deformities, especially in the developing countries. Furthermore, polysomnography may have the “first-night” effect (i.e., the result of the first exam), which may differ significantly from the results of subsequent exams after the patient gets adapted to it^24^. A high number of individuals with DFDs who sought surgical treatment and who, despite the presence of the symptoms of SED, had not yet been diagnosed with OSAS. This indicates that the association between DFDs and “silent” OSAS is frequent, but underdiagnosed^3^.

In the present study, patients were evaluated for the presence of DFD with surgical indications. None of the patients underwent SARME or MA for the treatment for OSAS. Basal polysomnography was performed in all patients as part of the UNIFESP craniomaxillofacial surgery outpatient care protocol. The distribution of demographic characteristics between the groups was homogeneous, with the SARME group presenting with a mean age of 30.32 years and a BMI of 26.45 kg/m^2^, while the MA group had a mean age of 31.30 years and a BMI of 26.05 kg/m^2^.

The analysis of the association between the eight cephalometric measurements and the four polysomnographic measurements was performed by Pearson's correlation coefficient. When the whole sample was analyzed, a moderate positive correlation between AHI and BMI was observed. In the study by Borges et al.^15^, no association between BMI and AHI was reported; on the other hand, other studies by Kubota et al.^25^ and Tufik et al.^26^ demonstrated an association between the two parameters.

Age is also defined as a risk factor for OSAS. However, in this study, the correlation obtained was not significant, perhaps because the overall mean age (30.79 years) was low and uniform between the two groups. Furthermore, AHI was positively associated with SNA (r = 0.499/p = 0.011) and MP-H (r = 0.573/p = 0.03) and negatively associated with UAS (r = − 0.488/p = 0.013) in the preoperative evaluation, with no significant associations with T2 measurements in the SARME groups. This finding is in agreement with the classic study by Guilleminault et al.^4^, and that by Armalaite et al.^27^. In addition, the association between AHI and MP-H has been reported by Borges et al.^15^, Silva et al.^28^ and Ryu et al.^29^.

The correlation between MP-H, UAS, and AHI would translate into a more caudally located larynx, which has been associated with a risk factor for OSAS, whereas a higher UAS would translate into a larger upper airway caliber and therefore, would be expected. Lower inspiratory collapse during sleep, with lower AHI. However, a more anterior maxilla would be expected to provide larger retropalatal airspace and, consequently, a smaller AHI. This finding was not verified in the current study. Unlike the study by Parhiz et al.^30^, the average initial SNA in the present study was 82.94, and reached 81.78 postoperatively. Bretos et al.^31^ observed maxillary displacement in the group that underwent SARME with Haas but not in the group that underwent SARME with Hyrax. This correlation, although significant, is not as evident in the scatter plot, and should be considered with caveats.

Arousals index was positively correlated with SNA (r = 0.410/p = 0.042), Co-A (r = 0.491/p = 0.013), and MPH (r = 0507/p = 0.010), preoperatively. Similarly, as with AHI, the translation of MP-H into the lower larynx predisposes to obstructive events with increased AHI, and consequently, to arousals. A positive correlation between SNA would not be expected, translating a more anterior maxilla and a greater number of arousals. Another unexpected positive correlation was between Co-A, effective maxillary length, and a higher rate of arousals. The validity of such correlations may be questioned because the variation in the average Co-A was insignificant between T1 and T2 (82.70–82.12). Nevertheless, the scatter plots in this study did not suggest a significant correlation. Such associations may have occurred in isolation in this sample, but the fact that the anteroposterior dimension of the maxilla may be correlated with obstructive events and arousals in patients with transverse maxillary deficiency warrants further evaluations. One might speculate that this enlarged anterioposterior dimension could result in a more constricted maxilla thus, justifying the obstructive events and arousals.

Changes in the lateral wall of the oropharynx and the narrowing of its caliber have already been suggested by Cahali et al.^32^ as a possible predisposing factor for OSAS, having served as the basis for the development of the lateral pharyngoplasty technique. Such changes in transverse length could also be associated with the development of obstructive sleep events in more cranially located anatomical sites, such as nasal cavities and nasopharynx.

Significant correlations were observed preoperatively in patients belonging to the MA group. AHI was positively correlated with CoA (r = 0.424/p = 0.044), PoOr-A (r = 0.551/p = 0.006), and MPH (r = 0.479/p = 0.021). In this case, an increase in the distance from point A to the Frankfurt line (an increase in the vertical dimension of the maxilla) was observed, which may translate into dolichofacial individuals and might be a predisposing factor for obstructive events, as suggested by Kubota et al.^25^. As mentioned previously, an increased MP-H, representing a lower-located larynx, was associated with obstructive events in patients with decreased anteroposterior maxillary dimensions. However, Co-A, which represents the effective maxillary length, was positively correlated with AHI (mean value, 83.46–88.93). Such a correlation was unexpected because its increase following surgery should have been positively correlated with the postoperative values since there was a slight increase in AHI between T1 and T2, but the persistence of this correlation was not observed. The trend line in the scatter plot is influenced by an outlier and that the other group together without such a clear correlation.

There was a negative correlation between the arousals index and the UAS (r = − 0.488/p = 0.013) ; therefore, the higher the index the lower the number of arousals per hour. Similar to the SARME group, a larger UAS provides a clearer airway without the propensity for collapse. Such an association might be expected with AHI, a fact that was not verified in this study.

The mean maxillary advancement was 7.02 mm, which was higher than that presented by Hellak et al.^33^. Furthermore, the average increase in UAS was 27.05% in the MA group; this is lower than that (37.70%) reported by Hernández-Alfaro et al.^34^. Likewise, Rosario et al.^35^ observed a significant increase in UAS volume, but question the sustainability of this increase in the long term.

Despite the use of modern technological imaging resources, the delimitation of anatomical points can prove challenging and may lead to inaccuracies in the method. Some anatomical landmarks such as Porion, Condylum, and Gonius have low reliability in the inter-observer analysis, regardless of the degree of experience of the examiner^36^. Although cephalometric measurements have good reproducibility, measurement errors may occur due to difficulties in identifying certain anatomical landmarks. The values of ICC for all the cephalometric variables in this study are above 0.96 and any value above 0.9 is considered to have excellent reliability^37^.

In the present study, the Sella (S), Nasion (N), Supramentonian (B), Hyoid (H), and Mento (Me) were relatively easy to identify. However, the other demarcated points may present with relative difficulties due to the presence of overlapping images and the rotation of the patient's head. Nonetheless, despite the limitations concerning the identification of the anatomical landmarks and the potential risk of variations in the interobserver measurements, cephalometry is a highly relevant method used in medical and dental practice because it is easily acquired, has low exposure to ionizing radiation, and is cost-effective. Furthermore, this method has gained importance in detecting the skeletal changes related to various conditions, such as OSAS, and its use has extended to the detection of areas of upper airway narrowing.

Finally, negative correlations were observed between SNA, SNB, Co-A, and the minimum O2 saturation; however, they were not evident in the scatter plots. The significant correlations obtained were of moderate degree; however, MP-H may prove to be a promising cephalometric measure that can be used to demonstrate an association with OSAS, as demonstrated by Hoekema et al.^38^. Further studies that associate such measures with validated questionnaires for OSAS or excessive daytime sleepiness (Berlin or Epworth, respectively) can be sought as a way to increase the predictive value for OSAS. Moreover, studies that, through multiple regression, seek mathematical equations that determine the predictive value of surgeries or the predictive value for OSAS detection, as performed by Kim et al.^39^ could provide relevant information.

## Conclusions

The findings of this study showed that BMI was correlated with AHI and arousals. In the SARME group, correlations between AHI and SNA, UAS and MP-H, awakenings and SNA, and Co-A and MP-H were observed. In the MA group, correlations between AHI and Co-A, PoOr-A and MP-H, awakenings and UAS, and between the minimum saturation of O2 and SNA, SNB, and Co-A were observed.

## Supplementary Information

Supplementary Information 1.

Supplementary Information 2.

## Abbreviations

AHI: Apnea Hypopnea Index
BMI: Body Mass Index
DFD: Dentofacial deformities
ICC: Intraclass correlation coefficient
LAS: Lower airspace
MA: Maxillary advancement
MP-H: Hyoid bone height
OSA: Obstructive sleep apnea
OSAS: Obstructive sleep apnea syndrome
SARME: Surgically assisted rapid maxillary expansion
SED: Snoring episode duration
SNA: Sella-nasion-point A
SNB: Sella-nasion-point B
UAS: Upper airspace
