# Supplementary material for: Cephalometric measures correlate with polysomnography parameters in individuals with midface deficiency
Source: Sci Rep. 2021 Apr 12;11:7949. doi: 10.1038/s41598-021-85935-7 (PMC8042057; doi:10.1038/s41598-021-85935-7)
Supplement: Supplementary file 1 — Supplementary Information 1. [file 41598_2021_85935_MOESM1_ESM.docx]

**Figure 2** – Scatter plot for AHI and SNA.

**Figure 3** – Scatter plot for AHI and UAS.

**Figure 4** - Scatter plot for AHI and MP-H.

**Figure 5** - Scatter plot for Arousals and SNA.

**Figure 6**- Scatter plot for Arousals and Co-A.

**Figure 7** - Scatter plot for Arousals e MP-H.

**Figure 8** - Scatter plot for AHI and Co-A.

**Figure 9** – Scatter plot for AHI and PoOr-A.

**Figure 10** - Scatter plot for AHI and MP-H.

**Figure 11** – Scatter plot for Arousal and UAS.

**Figure 12** – Scatter plot for MinSatO2 and SNA.

**Figure 13** – Scatter plot for MinSatO2 and SNB.

**Figure 14** – Scatter plot for MinSatO2 and Co-A
