# Supplementary material for: Cephalometric measures correlate with polysomnography parameters in individuals with midface deficiency
Source: Sci Rep. 2021 Apr 12;11:7949. doi: 10.1038/s41598-021-85935-7 (PMC8042057; doi:10.1038/s41598-021-85935-7)
Supplement: Supplementary file 2 — Supplementary Information 2. [file 41598_2021_85935_MOESM2_ESM.docx]

**Online Supplementary Table 1.** Cephalometric measurements of patients undergoing SARME at T1 (preoperative) and T2 (6 months after surgery).

| **Patient** | **SNA T1** | **SNA T2** | **SNB T1** | **SNB T2** | **Co-A T1** | **Co-A T2** | **PoOr-A T1** | **PoOr-A T2** | **NPerp- A**  **T1** | **NPerp-A**  **T2** | **UAS T1** | **UAS T2** | **IAS T1** | **IAS T2** | **MP-H T1** | **MP-H T2** |
| --- | --- | --- | --- | --- | --- | --- | --- | --- | --- | --- | --- | --- | --- | --- | --- | --- |
| 1 | 85,37 | 83,77 | 84,36 | 84,07 | 87,21 | 82,47 | 36,24 | 32,41 | 1,80 | 2,05 | 6,70 | 8,14 | 7,39 | 6,11 | 9,43 | 13,26 |
| 2 | 84,62 | 80,83 | 77,94 | 74,98 | 81,36 | 79,74 | 32,92 | 26,40 | 1,33 | −1,74 | 6,07 | 6,71 | 2,66 | 7,93 | 8,55 | 12,97 |
| 3 | 84,11 | 81,60 | 76,10 | 76,90 | 84,46 | 74,84 | 28,71 | 25,52 | −2,40 | −2,34 | 10,48 | 9,79 | 7,17 | 16,75 | 16,80 | 18,71 |
| 4 | 80,89 | 82,33 | 72,65 | 74,18 | 75,18 | 76,92 | 26,12 | 28,06 | 0,65 | 0,10 | 10,22 | 11,51 | 6,39 | 12,97 | 10,82 | 14,68 |
| 5 | 86,29 | 87,35 | 83,12 | 83,72 | 86,51 | 84,43 | 27,38 | 26,83 | −1,79 | 1,47 | 8,06 | 7,82 | 9,81 | 5,87 | 9,81 | 9,63 |
| 6 | 84,62 | 86,20 | 80,92 | 81,15 | 86,44 | 90,34 | 29,17 | 31,27 | 0,50 | 2,65 | 10,71 | 8,28 | 11,84 | 7,63 | 9,86 | 7,60 |
| 7 | 87,86 | 84,99 | 78,32 | 75,72 | 80,45 | 78,17 | 25,85 | 28,42 | 0,31 | 0,25 | 5,24 | 8,15 | 11,11 | 4,21 | 19,70 | 20,85 |
| 8 | 83,29 | 81,30 | 82,27 | 79,45 | 91,95 | 91,24 | 36,01 | 27,95 | 0,56 | 1,96 | 10,35 | 8,64 | 12,79 | 14,71 | 23,99 | 25,51 |
| 9 | 74,32 | 73,14 | 78,92 | 79,78 | 81,35 | 80,19 | 28,82 | 26,34 | −3,69 | −5,05 | 8,35 | 9,27 | 10,33 | 11,15 | 18,04 | 16,18 |
| 10 | 85,38 | 84,99 | 82,89 | 83,33 | 85,37 | 83,86 | 25,57 | 24,51 | 0,22 | 1,56 | 10,97 | 8,12 | 9,65 | 8,67 | 19,04 | 21,27 |
| 11 | 83,77 | 83,70 | 78,64 | 79,14 | 83,88 | 87,50 | 29,55 | 34,58 | 6,13 | 3,66 | 7,24 | 9,77 | 7,27 | 8,34 | 19,39 | 14,87 |
| 12 | 83,43 | 83,66 | 82,24 | 81,18 | 85,85 | 83,06 | 22,36 | 21,87 | −1,74 | −5,73 | 10,78 | 11,48 | 11,59 | 16,18 | 16,65 | 15,16 |
| 13 | 77,02 | 77,81 | 75,21 | 75,45 | 83,10 | 86,59 | 32,02 | 29,95 | 2,46 | 2,99 | 9,02 | 6,02 | 11,98 | 12,36 | 17,50 | 19,33 |
| 14 | 78,57 | 78,71 | 73,36 | 72,64 | 75,70 | 70,98 | 29,01 | 26,17 | 2,09 | 0,74 | 6,14 | 6,78 | 6,16 | 11,97 | 23,05 | 29,37 |
| 15 | 87,18 | 78,86 | 87,63 | 77,68 | 75,17 | 79,33 | 24,46 | 29,41 | 2,06 | 4,26 | 15,91 | 5,52 | 19,90 | 12,48 | 18,62 | 20,15 |
| 16 | 83,83 | 77,71 | 81,77 | 74,28 | 79,61 | 79,50 | 25,27 | 25,46 | 1,13 | 1,73 | 10,64 | 12,41 | 4,49 | 9,79 | 17,90 | 19,34 |
| 17 | 84,09 | 82,40 | 81,52 | 80,18 | 82,23 | 81,77 | 21,88 | 25,25 | −5,09 | 3,30 | 10,75 | 7,58 | 4,61 | 6,11 | 11,89 | 14,91 |
| 18 | 79,77 | 81,09 | 77,77 | 78,36 | 83,81 | 84,72 | 26,81 | 24,29 | −3,86 | −5,97 | 13,47 | 13,88 | 7,89 | 6,21 | 6,83 | 1,88 |
| 19 | 86,32 | 83,57 | 91,48 | 89,20 | 83,03 | 84,56 | 27,03 | 29,45 | −6,11 | −5,07 | 15,00 | 15,35 | 21,62 | 18,45 | 20,99 | 14,59 |
| 20 | 92,05 | 89,22 | 86,33 | 86,22 | 87,38 | 82,68 | 26,95 | 26,66 | −5,91 | −5,97 | 3,80 | 3,83 | 12,17 | 11,23 | 30,07 | 24,24 |
| 21 | 81,10 | 81,05 | 75,45 | 76,19 | 86,48 | 88,61 | 28,58 | 32,29 | −8,17 | −5,50 | 12,23 | 11,57 | 13,42 | 8,14 | 18,74 | 16,09 |
| 22 | 82,36 | 81,25 | 81,32 | 81,60 | 79,43 | 77,79 | 30,85 | 26,83 | 1,75 | 1,97 | 8,39 | 8,69 | 14,00 | 11,21 | 13,14 | 14,96 |
| 23 | 76,18 | 76,40 | 81,06 | 82,38 | 75,88 | 75,76 | 21,93 | 19,77 | 5,76 | 1,76 | 7,88 | 14,02 | 7,25 | 11,79 | 11,67 | 17,14 |
| 24 | 80,14 | 80,50 | 78,25 | 78,50 | 80,85 | 81,91 | 19,69 | 22,04 | −7,00 | −6,68 | 4,18 | 4,96 | 7,83 | 8,54 | 23,28 | 20,62 |
| 25 | 80,98 | 82,12 | 77,98 | 77,36 | 84,95 | 86,16 | 24,76 | 19,12 | −6,35 | −6,21 | 6,30 | 8,17 | 5,65 | 6,17 | 12,23 | 19,35 |
| **Average** | 82,94 | 81,78 | 80,30 | 79,35 | 82,70 | 82,12 | 27,52 | 26,83 | −1,01 | −0,79 | 9,16 | 9,06 | 9,80 | 10,20 | 16,32 | 16,91 |

T1: preoperative; T2: postoperative; SNA and SNB: degrees; Co-A, PoOr-A, NPerp-A, UAS, IAS, MP-H: millimeters.

**Online Supplementary Table 2.** Values of T1 (preoperative) and T2 (6 months after surgery) polysomnographic measurements in patients who underwent SARME.

| **Patient** | **AHI T1** | **AHI T2** | **Arousals T1** | **Arousals T2** | **MinSatO2 T1** | **MinSatO2 T2** | **TST sat<90% T1** | **TST sat<90% T2** |
| --- | --- | --- | --- | --- | --- | --- | --- | --- |
| 1 | 3,00 | 3,30 | 0,00 | 10,95 | 89,00 | 94,00 | 3,20 | 0,00 |
| 2 | 0,90 | 0,00 | 0,00 | 0,00 | 94,00 | 98,00 | 0,00 | 0,00 |
| 3 | 0,20 | 0,00 | 12,00 | 0,80 | 69,00 | 93,00 | 0,30 | 0,00 |
| 4 | 1,30 | 0,00 | 0,00 | 5,80 | 89,00 | 92,00 | 0,10 | 0,00 |
| 5 | 13,40 | 27,30 | 0,00 | 0,00 | 85,00 | 63,00 | 0,40 | 7,40 |
| 6 | 0,40 | 0,20 | 14,00 | 16,50 | 95,00 | 96,00 | 0,00 | 0,00 |
| 7 | 18,30 | 6,80 | 6,90 | 38,30 | 61,00 | 83,00 | 6,10 | 0,50 |
| 8 | 8,10 | 10,80 | 25,00 | 19,10 | 91,00 | 89,00 | 0,00 | 0,20 |
| 9 | 3,00 | 4,30 | 0,00 | 23,00 | 86,00 | 87,00 | 3,20 | 3,00 |
| 10 | 13,20 | 10,02 | 15,00 | 11,44 | 71,00 | 87,00 | 3,10 | 5,80 |
| 11 | 0,00 | 0,00 | 0,00 | 0,90 | 96,00 | 94,00 | 0,00 | 0,00 |
| 12 | 0,10 | 1,20 | 8,00 | 4,00 | 95,00 | 95,00 | 0,00 | 0,00 |
| 13 | 0,30 | 0,30 | 12,00 | 15,00 | 92,00 | 92,00 | 0,00 | 0,00 |
| 14 | 1,20 | 0,10 | 0,00 | 0,00 | 92,00 | 95,00 | 0,00 | 0,00 |
| 15 | 0,40 | 0,00 | 0,00 | 18,40 | 93,00 | 95,00 | 0,00 | 0,00 |
| 16 | 2,00 | 3,20 | 9,00 | 11,20 | 92,00 | 91,00 | 0,00 | 0,00 |
| 17 | 1,00 | 0,60 | 9,90 | 5,20 | 97,00 | 95,00 | 0,00 | 0,00 |
| 18 | 2,60 | 2,40 | 7,00 | 9,40 | 95,00 | 91,00 | 0,00 | 0,00 |
| 19 | 0,20 | 1,70 | 0,00 | 11,60 | 93,00 | 94,00 | 0,00 | 0,00 |
| 20 | 44,80 | 15,00 | 42,70 | 24,90 | 85,00 | 84,00 | 4,30 | 2,10 |
| 21 | 6,50 | 2,97 | 0,00 | 23,11 | 67,00 | 92,00 | 3,80 | 0,00 |
| 22 | 0,00 | 0,00 | 0,00 | 0,00 | 91,00 | 93,00 | 0,00 | 0,00 |
| 23 | 0,00 | 0,50 | 4,40 | 0,00 | 93,00 | 93,00 | 0,00 | 0,00 |
| 24 | 20,70 | 23,60 | 6,00 | 25,10 | 76,00 | 73,00 | 1,30 | 4,90 |
| 25 | 0,30 | 1,40 | 2,70 | 0,00 | 93,00 | 81,00 | 0,00 | 1,10 |
| **Average** | 5,68 | 4,63 | 6,98 | 10,99 | 87,20 | 89,60 | 1,03 | 1,00 |

AHI: number per hour; MinSatO2: percentage; TST sat<90%: percentage of the total time of sleep.

**Online Supplementary Table 3.** Cephalometric measurements at T1 (preoperative) and T2 (6 months after surgery) in patients who underwent MA.

| **Patient** | **SNA T1** | **SNA T2** | **SNB T1** | **SNB T2** | **Co-A T1** | **Co-A T2** | **PoOr-A T1** | **PoOr-A T2** | **NPerp-A**  **T1** | **NPerp-A**  **T2** | **UAS T1** | **UAS T2** | **IAS T1** | **IAS T2** | **MP-H T1** | **MP-H T2** |
| --- | --- | --- | --- | --- | --- | --- | --- | --- | --- | --- | --- | --- | --- | --- | --- | --- |
| 1 | 89,75 | 97,93 | 89,83 | 93,98 | 94,13 | 102,27 | 28,16 | 28,36 | 2,12 | −10,85 | 8,55 | 11,43 | 11,26 | 7,17 | 23,37 | 16,13 |
| 2 | 80,23 | 83,70 | 81,48 | 79,82 | 53,13 | 85,07 | 16,02 | 23,70 | 1,87 | −4,20 | 6,21 | 13,47 | 9,79 | 12,45 | 8,95 | 11,67 |
| 3 | 81,33 | 84,34 | 84,72 | 82,70 | 88,08 | 92,47 | 32,03 | 33,32 | 1,35 | −6,48 | 10,82 | 16,69 | 14,84 | 8,09 | 16,65 | 17,74 |
| 4 | 83,87 | 90,15 | 80,50 | 83,60 | 81,46 | 85,96 | 31,01 | 30,21 | 0,39 | −11,81 | 4,81 | 9,68 | 6,47 | 10,54 | 14,54 | 13,57 |
| 5 | 86,96 | 92,62 | 86,47 | 85,43 | 79,47 | 88,25 | 25,50 | 29,18 | 0,76 | −5,86 | 8,37 | 13,19 | 6,12 | 12,30 | 12,39 | 8,69 |
| 6 | 76,36 | 84,52 | 80,35 | 81,76 | 81,53 | 83,39 | 34,98 | 28,07 | 4,88 | −5,12 | 11,13 | 14,03 | 9,77 | 12,52 | 12,30 | 11,52 |
| 7 | 81,52 | 91,84 | 83,57 | 89,13 | 83,30 | 85,59 | 29,32 | 32,28 | 1,55 | −1,54 | 8,71 | 11,61 | 9,19 | 8,33 | 8,79 | 6,57 |
| 8 | 84,54 | 90,82 | 90,50 | 88,78 | 90,87 | 98,29 | 28,53 | 29,21 | 0,81 | −10,66 | 20,37 | 21,19 | 15,62 | 13,52 | 7,52 | 5,81 |
| 9 | 87,63 | 93,66 | 94,89 | 92,66 | 86,28 | 95,70 | 26,68 | 29,51 | 2,51 | −9,09 | 13,93 | 19,97 | 10,13 | 18,30 | 5,33 | 9,02 |
| 10 | 82,78 | 87,82 | 84,65 | 84,36 | 86,44 | 90,73 | 28,34 | 26,70 | 3,64 | −6,11 | 14,05 | 18,04 | 21,36 | 10,59 | 7,07 | 9,41 |
| 11 | 74,50 | 84,18 | 80,87 | 82,74 | 92,46 | 94,50 | 37,28 | 36,64 | 7,63 | −3,03 | 9,05 | 16,65 | 9,14 | 10,51 | 12,69 | 8,56 |
| 12 | 82,39 | 98,28 | 89,72 | 89,78 | 77,80 | 92,82 | 25,78 | 28,11 | 1,41 | −13,18 | 10,35 | 11,70 | 11,83 | 7,89 | 6,45 | 6,96 |
| 13 | 92,30 | 98,06 | 94,20 | 94,95 | 91,83 | 97,75 | 32,52 | 31,58 | −3,81 | −12,61 | 13,67 | 16,59 | 18,70 | 16,96 | 13,51 | 13,66 |
| 14 | 78,85 | 87,95 | 80,64 | 82,91 | 80,90 | 81,64 | 27,94 | 26,64 | −4,20 | −7,84 | 6,86 | 7,66 | 9,99 | 6,39 | 11,44 | 9,80 |
| 15 | 90,04 | 92,85 | 90,45 | 89,32 | 77,76 | 57,19 | 26,32 | 19,93 | −7,53 | −4,08 | 12,46 | 9,21 | 12,27 | 9,07 | 9,37 | 5,51 |
| 16 | 78,15 | 81,67 | 81,17 | 81,35 | 86,39 | 91,02 | 32,71 | 30,50 | 7,22 | −1,29 | 12,70 | 12,61 | 18,34 | 16,99 | 24,49 | 20,29 |
| 17 | 79,92 | 86,90 | 86,87 | 85,44 | 80,44 | 92,33 | 24,49 | 25,39 | 0,77 | −6,66 | 12,98 | 14,96 | 9,00 | 12,32 | 15,72 | 16,53 |
| 18 | 87,49 | 91,37 | 91,54 | 88,22 | 90,02 | 96,53 | 25,75 | 31,23 | −1,27 | −4,56 | 10,85 | 17,02 | 12,18 | 19,07 | 8,46 | 7,16 |
| 19 | 79,71 | 83,34 | 80,97 | 79,19 | 86,76 | 81,89 | 26,18 | 27,71 | 1,87 | −5,08 | 12,12 | 10,96 | 11,80 | 12,44 | 12,21 | 10,45 |
| 20 | 77,41 | 88,13 | 85,52 | 84,30 | 77,50 | 86,86 | 25,17 | 29,56 | 3,39 | −5,81 | 9,70 | 13,25 | 13,19 | 12,98 | 17,68 | 16,90 |
| 21 | 85,08 | 89,87 | 86,10 | 84,19 | 85,32 | 92,92 | 29,04 | 34,06 | 0,56 | −8,16 | 12,34 | 17,63 | 8,35 | 16,62 | 14,22 | 14,18 |
| 22 | 79,60 | 82,08 | 78,52 | 75,73 | 80,53 | 86,56 | 29,14 | 31,44 | 3,49 | −8,95 | 14,19 | 15,95 | 7,28 | 9,63 | 13,04 | 15,76 |
| 23 | 78,35 | 83,18 | 81,46 | 80,85 | 87,12 | 85,59 | 25,52 | 22,40 | 0,76 | −6,82 | 11,51 | 13,90 | 11,33 | 14,51 | 15,11 | 13,04 |
| **Average** | 82,55 | 88,92 | 85,43 | 85,27 | 83,46 | 88,93 | 28,19 | 28,95 | 1,31 | −6,95 | 11,12 | 14,23 | 11,65 | 12,14 | 12,66 | 11,69 |

SNA and SNB: degrees; Co-A, PoOr-A, NPerp-A, UAS, IAS, MP-H: millimeters.

**Online Supplementary Table 4.** Values of T1 (preoperative) and T2 (6 months after surgery) polysomnographic measurements in patients who underwent MA.

| **Patient** | **AHI T1** | **AHI T2** | **Arousals T1** | **Arousals T2** | **MinSatO2 T1** | **MinSatO2 T2** | **TST sat<90% T1** | **TST sat<90% T2** |
| --- | --- | --- | --- | --- | --- | --- | --- | --- |
| 1 | 10,70 | 9,10 | 17,00 | 22,30 | 90,00 | 88,00 | 0,00 | 22,30 |
| 2 | 0,20 | 0,70 | 25,00 | 9,00 | 97,00 | 94,00 | 0,00 | 9,00 |
| 3 | 2,40 | 2,36 | 2,40 | 17,00 | 95,00 | 92,00 | 0,00 | 17,00 |
| 4 | 0,60 | 0,00 | 18,00 | 12,00 | 94,00 | 93,00 | 0,00 | 12,00 |
| 5 | 2,10 | 1,30 | 13,00 | 4,70 | 86,00 | 92,00 | 3,20 | 4,70 |
| 6 | 0,20 | 0,60 | 0,40 | 11,70 | 95,00 | 89,00 | 0,00 | 11,70 |
| 7 | 0,50 | 0,00 | 4,70 | 4,40 | 93,00 | 92,00 | 0,00 | 4,40 |
| 8 | 0,00 | 0,20 | 7,50 | 6,60 | 94,00 | 94,00 | 0,00 | 6,60 |
| 9 | 2,20 | 7,60 | 7,80 | 3,10 | 85,00 | 86,00 | 1,20 | 3,10 |
| 10 | 0,00 | 2,10 | 5,70 | 2,20 | 92,00 | 91,00 | 0,00 | 2,20 |
| 11 | 18,00 | 33,40 | 45,00 | 30,00 | 91,00 | 72,00 | 0,00 | 30,00 |
| 12 | 0,00 | 0,00 | 15,00 | 11,00 | 98,00 | 95,00 | 0,00 | 11,00 |
| 13 | 11,20 | 6,80 | 22,20 | 13,20 | 84,00 | 84,00 | 0,50 | 13,20 |
| 14 | 4,00 | 0,70 | 21,20 | 9,70 | 95,00 | 94,00 | 0,00 | 9,70 |
| 15 | 0,00 | 4,20 | 2,50 | 11,20 | 97,00 | 91,00 | 0,00 | 11,20 |
| 16 | 11,40 | 6,10 | 13,10 | 9,70 | 88,00 | 92,00 | 2,50 | 9,70 |
| 17 | 0,50 | 2,50 | 0,00 | 7,70 | 91,00 | 93,00 | 0,00 | 7,70 |
| 18 | 0,00 | 1,10 | 6,90 | 3,70 | 92,00 | 90,00 | 0,00 | 3,70 |
| 19 | 0,00 | 0,90 | 7,90 | 11,20 | 94,00 | 92,00 | 0,00 | 11,20 |
| 20 | 1,70 | 1,10 | 8,20 | 11,50 | 93,00 | 93,00 | 0,00 | 11,50 |
| 21 | 0,00 | 0,50 | 4,80 | 3,80 | 94,00 | 92,00 | 0,00 | 3,80 |
| 22 | 0,00 | 0,20 | 0,00 | 0,00 | 97,00 | 93,00 | 0,00 | 0,00 |
| 23 | 0,00 | 0,60 | 11,20 | 8,40 | 97,00 | 93,00 | 0,00 | 8,40 |
| **Average** | 2,86 | 3,57 | 11,28 | 9,74 | 92,70 | 90,65 | 0,32 | 9,74 |

AHI: number per hour; MinSatO2: percentage; TST sat<90%: percentage of the total time of sleep.
